# Correction: Infusing behavior science into large language models for activity coaching

**DOI:** 10.1371/journal.pdig.0000786

**Published:** 2025-03-03

**Authors:** Madhurima Vardhan, Narayan Hegde, Deepak Nathani, Emily Rosenzweig, Cathy Speed, Alan Karthikesalingam, Martin Seneviratne

The authors are listed out of order. Please view the correct author order, affiliations, and citation here:

Madhurima Vardhan^1^, Narayan Hegde^1^, Deepak Nathani^1^, Emily Rosenzweig^2^, Cathy Speed^3^, Alan Karthikesalingam^3^, Martin Seneviratne^3^

**1** Google Research, Bangalore, India, **2** Verily Life Sciences, San Francisco, United States of America, **3** Google Health, London, United Kingdom

Vardhan M, Hegde N, Nathani D, Rosenzweig E, Speed C, Karthikesalingam A, et al. (2024) Infusing behavior science into large language models for activity coaching. PLOS Digit Health 3(4): e0000431. https://doi.org/10.1371/journal.pdig.0000431

There are errors in the Author Contributions. Conceptualization MV, NH, Data curation MV, NH, Formal analysis MV, NH, Investigation MV, NH, Methodology MV, NH, Project Administration MV, NH, Validation MV, NH, Visualization MV, NH, Writing – Original Draft Preparation MV, NH.
