# Supplementary material for: Infusing behavior science into large language models for activity coaching
Source: PLOS Digit Health. 2024 Apr 2;3(4):e0000431. doi: 10.1371/journal.pdig.0000431 (PMC10986996; doi:10.1371/journal.pdig.0000431)
Supplement: S1 Table — (PDF) [file pdig.0000431.s001.pdf]

**Bleu Score**	**1-gram**	**2-gram**	**3-gram**	**4-gram**
Non Primed	7.31	4.24	2.57	1.78
Primed using randomly selected 30 coach sentences from PACE study	17.89	8.52	5	3.34
Primed with coach crafted 30 sentences for PACE study	22.312	10.894	5.356	3.289

S1 Table : BLEU(BiLingual Evaluation Understudy) match score to compare LLM primiring strategies to match human coach sentences.
