# Supplementary material for: Infusing behavior science into large language models for activity coaching
Source: PLOS Digit Health. 2024 Apr 2;3(4):e0000431. doi: 10.1371/journal.pdig.0000431 (PMC10986996; doi:10.1371/journal.pdig.0000431)
Supplement: S2 Table — (PDF) [file pdig.0000431.s002.pdf]

Index	Category	User query	Days
1	**Motivation**	I was able to meet my goal of step count in yesterday's walk, but sometimes it is hard to meet the goal.	7
2	**Motivation**	I would be more inclined to go walking if it could be a bit more fun.	5,8
3	**Motivation**	I am busy today and not sure of all the benefits of daily walking on health and well-being.	2,4
4	**Capability**	Hi coach, unfortunately I have injured myself. I can't walk today.	5,11
5	**Capability**	I really don't think it is the best use of time. I just don't think it would be worth it.	9
6	**Capability**	Hi coach, it is raining outside. I do not want to walk in the rain and I do not have a treadmill. Is there anything else I can do?	6,2
7	**Opportunity**	I am super busy with work today.I have chores to do in the morning and work meetings after that.	1,7,5
8	**Opportunity**	Hi coach, sure but I am not able to get it in my routine. How can I plan better ?	4,2,8
9	**Opportunity**	Hi Coach, 30 minutes would be a lot. Is there any other alternative you could suggest?	8,8

S2 Table : User queries across COM [Capability, Opportunity and Motivation] themes selected for Large Language Model evaluation. Days column represent the days when the query was asked by different users to coaches in the PACE study
