# Supplementary material for: Infusing behavior science into large language models for activity coaching
Source: PLOS Digit Health. 2024 Apr 2;3(4):e0000431. doi: 10.1371/journal.pdig.0000431 (PMC10986996; doi:10.1371/journal.pdig.0000431)
Supplement: S1 Text — (PDF) [file pdig.0000431.s003.pdf]

Coach Phrase Priming sentence selection method 30 sentences crafted by 6 fitness experts were used for priming Large Language Models (LLMs) consisting of 10 sentences from capability, opportunity and motivation (COM) themes according to the COM-B model. These sentences were crafted to cater most common fitness coaching related queries experienced by the coaches. The handcrafted messages were provided as template conversations in the conversation tool for the WOO arm in the PACE study. The PACE algorithm selected a suitable template as response to user query according to the FBM model. The coaches were free to copy as is or modify the template or write their own response in the wizard of Oz setup. The efficacy of the sentences used in priming were tested using the bleu score by matching the coach responses collected from the PACE study and output generated by the unprimed/primed LLMs. The S1 Table describes the bleu match score LLM response with or without priming. The BLEU score (BiLingual Evaluation Understudy) is a metric for comparing similarity between a query and reference text based on matching n-grams. The sentences selected for the coach phrase priming have equal representation of motivation, opportunity and capability messages. These carefully selected sentences caused the LLMs output to have the highest match with the coach utterances in the PACE study as measured by the BLEU score. The order of the 30 selected sentences used in priming influences the LLMs output as shown in the previous works[ref]. When the sentences used to prime are batched by the theme (COM), then the output is biased towards the last theme. We randomly order the sentences mixing all three themes but the order is kept consistent when priming for each conversation for reproducibility. Priming based on one of the COM-B themes narrows the LLM output and the quality as measured qualitatively. Since the user query can span across a wide range of fitness issues and straddle multiple COM themes, we do not prime the LLMs with any single predetermined themed sentences. Rather, a custom re-ranking algorithm described later is used for tighter control of the COM-theme primed LLMs output to cater to the specific user query.
