# Supplementary material for: Infusing behavior science into large language models for activity coaching
Source: PLOS Digit Health. 2024 Apr 2;3(4):e0000431. doi: 10.1371/journal.pdig.0000431 (PMC10986996; doi:10.1371/journal.pdig.0000431)
Supplement: S2 Text — (PDF) [file pdig.0000431.s004.pdf]

Large Language Model (LLM) based conversation evaluation tool and methodology

A conversational simulator (S2 Fig) to display the coach and user conversation session was developed to test the efficacy of the proposed LLM enhancements. Each conversation session in the simulator consists of a series of dialogue exchanges between user and LLM to resolve a specific user generated fitness issue. The conversation tool prompts the following message to initiate the conversation “Hey John, It's time for your morning walk.” A preselected user query is given to LLM which sets the conversation topic on a fitness related issue. 8 independent raters assess the quality of the LLMs response conditioned on the user query. The 9 user queries that were selected to test the LLM response as shown in supplementary table 2. These queries were not used in the training of user query classifiers. Three user queries from each of the COM themes were identified from various points in the course of the 21 day PACE study. These queries were the most commonly occurring user initiated conversations for each of the themes from the PACE study. The participants in the pace study were free to pick any walking related questions to ask the coaches. This evaluation strategy allows testing the LLM response on important user queries which may arise after multiple days of interaction with coaching assistants which is not possible if LLM are evaluated by having a one off conversation with a new participant each time. Also, having independent annotators to rate the conversation session helps reduce bias and also scale the number of evaluations to statistically significant numbers. Any follow up questions to the LLM response were added appropriately to continue the conversation on the original topic until a logical end is reached. 8 raters assessed the LLM response qualitatively for a given user query by answering 8 survey questions. The raters had an equal mix of age, gender and demographics without any prior fitness coaching experience. Raters were not incentivised for this exercise and were blinded to the LLM variation which generated the automated response and to the LLM technology. Raters used survey forms to submit the rating for each set of user - LLM conversation and were free to change the ratings at any time during the exercise.
