# Supplementary material for: Infusing behavior science into large language models for activity coaching
Source: PLOS Digit Health. 2024 Apr 2;3(4):e0000431. doi: 10.1371/journal.pdig.0000431 (PMC10986996; doi:10.1371/journal.pdig.0000431)
Supplement: S3 Text — (PDF) [file pdig.0000431.s005.pdf]

Coach response & User query classifier

To provide context relevant responses with right behavior science backed construct, it is important to recognize the topic queried by the user on walking and respond back on the same theme to address them and resolve the issue to enable walk more. Though LLMs are trained on a wide corpus and do reasonably well, there is potential to improve further, since LLMs do not explicitly understand behavior science constructs like motivation, capability, & opportunity nor have any in-built coaching specific classifier to determine user query topic. Given a user query sentence, the *user-query* classifier assigns high vs low value for each of the motivation, capability & opportunity (COM) dimensions. The COM-B model is used to pick the appropriate theme for coach response based on output values for COM dimensions as shown in figure 2 in methods. COM-B policy prioritizes addressing the category with low value and using the priority order : capability, opportunity and motivation to resolve any conflicts. PACE study showed ~95% of the user query could be classified as either high/low on atleast one of COM with confidence. Post action selection, the Reranking algorithm is used to modify LLM response to suit the selected COM theme.

The Reranking method builds on top of primed LLM which generates richer conversation responses. LLMs generate many candidates and stack rank using in-built logic developed in the training process. They fire the top-1 as the response to the user query. LLMs can be personalized further for fitness for coaching by recognizing the user query topic/theme and using it to re-rank the generated sentences by primed LLMs to match the user query theme based on COM-B policy. For example, if the user seeks alternatives to walking during rain, ReRanked LLMs recognize the user theme as lack of capability, and use the information to re-rank the capability enhancing messages relative to motivation and opportunity as suggested by the COM-B algorithm. Success of the method hinges on developing sufficiently accurate NLP based user query and LLM response classifiers to identify one of the COM themes for each input sentence.
