# Supplementary material for: Infusing behavior science into large language models for activity coaching
Source: PLOS Digit Health. 2024 Apr 2;3(4):e0000431. doi: 10.1371/journal.pdig.0000431 (PMC10986996; doi:10.1371/journal.pdig.0000431)
Supplement: S4 Text — (PDF) [file pdig.0000431.s006.pdf]

Summary of PACE study and adaptation to FIT-LLM work

The PACE coaching conversation tool was built to test the efficacy of automated conversational assistants in comparison to fitness experts to assist participants to improve walking habits through text based conversations on digital platforms as described in the paper. The Wizard of Oz (WOO) or treatment group had coaching conversations selected using COM-B policy to match user query. The PACE tool consisted of the coaching message repository crafted by the fitness experts and the COM-B algorithm to select the theme of the response to the user query. Coaches are free to use the suggested message by the tool, edit them or craft a new response on their own. Coaches went with the PACE tool suggested response in 80%+ cases and the efficacy of the WOO arm was comparable to fitness coach led control arm in both the qualitative and quantitative metrics discussed in the PACE paper. 520 conversations were generated using the PACE tool to assist 16 participants for the duration of 14 days on a wide variety of fitness related topics. A total of 6 independent annotators labeled these conversations as one of Motivation, Capability and Opportunity.A conversation session is usually on a single fitness topic but may have many pairs of user & coach dialogue exchanges leading to multiple training examples. This serves as a train & test set for respective classifiers. These conversations were used to train and evaluate coach response and user query theme classifier. Further, user queries with pre-defined criteria were selected to evaluate the LLMs post priming and re-ranking. This method led to selection of the most commonly asked user queries to test efficacy of LLMs on a wide variety of fitness related topics. The BERT is used as a base model to fine tune for user query and coach response COM classifiers. Weights are not shared between the models and are trained independently. The last classification layer is re-trained with cross entropy loss to develop a 3-way classifier to classify any input sentence as one of C/O/M.
