# Supplementary material for: Infusing behavior science into large language models for activity coaching
Source: PLOS Digit Health. 2024 Apr 2;3(4):e0000431. doi: 10.1371/journal.pdig.0000431 (PMC10986996; doi:10.1371/journal.pdig.0000431)
Supplement: S1 Fig — (PDF) [file pdig.0000431.s007.pdf]

A	
Coach	Hey John, It's time for your morning walk.
Human	Hi Coach - Yes, it is. I would more inclined to go if it could be a bit more fun.
Coach	That's a great attitude, John! Let's see if we can make your walk more fun. Maybe you could walk through a park or by a river. Second, let's try listening to some music or an audio book while you walk. Finally, let's see if we can get a friend or family member to join you on your walk.
Human	That sounds like a great plan! I'm going to go ask my wife if she wants to join me on my walk today. Thanks for the motivation, Coach!

B	
Coach	Hey John, It's time for your morning walk.
Human	Hi Coach - Yes, it is. I would more inclined to go if it could be a bit more fun.
Coach	Well, let's make it more fun then! Why don't we listen to some music or a podcast that you enjoy while we walk?
Human	That could probably help. But I still get bored alone.
Coach	How about we walk with a friend then?
Human	Maybe that could make walking less boring and more fun.
Coach	Let's try it and see!
Human	Thank you for the suggestion coach.
Coach	You're welcome! Let me know how it goes.

ID	Question	A	B
1	Which conversation provides a better overall coaching experience - A, B or unsure	B	
3	The coach provides concrete fitness strategies that are actionable to the user (1 - strongly disagree ⟶ 5- strongly agree)	5	5
4	The coach responds to user questions in a realistic manner (1 - strongly disagree ⟶ 5- strongly agree)	2	4
5	The coach provides motivation or encouragement to the user (1 - strongly disagree ⟶ 5- strongly agree)	4	4
6	The coach is empathetic toward the user's needs and challenges (1 - strongly disagree ⟶ 5- strongly agree)	4	4
7	The language used by the coach is realistic and appropriate for the setting (1 - strongly disagree ⟶ 5- strongly agree)	2	4

S1 Fig : LLM Conversation rating tool used by annotators
