# Supplementary figures and images for: Infusing behavior science into large language models for activity coaching
Source: PLOS Digit Health. 2024 Apr 2;3(4):e0000431. doi: 10.1371/journal.pdig.0000431 (PMC10986996; doi:10.1371/journal.pdig.0000431)

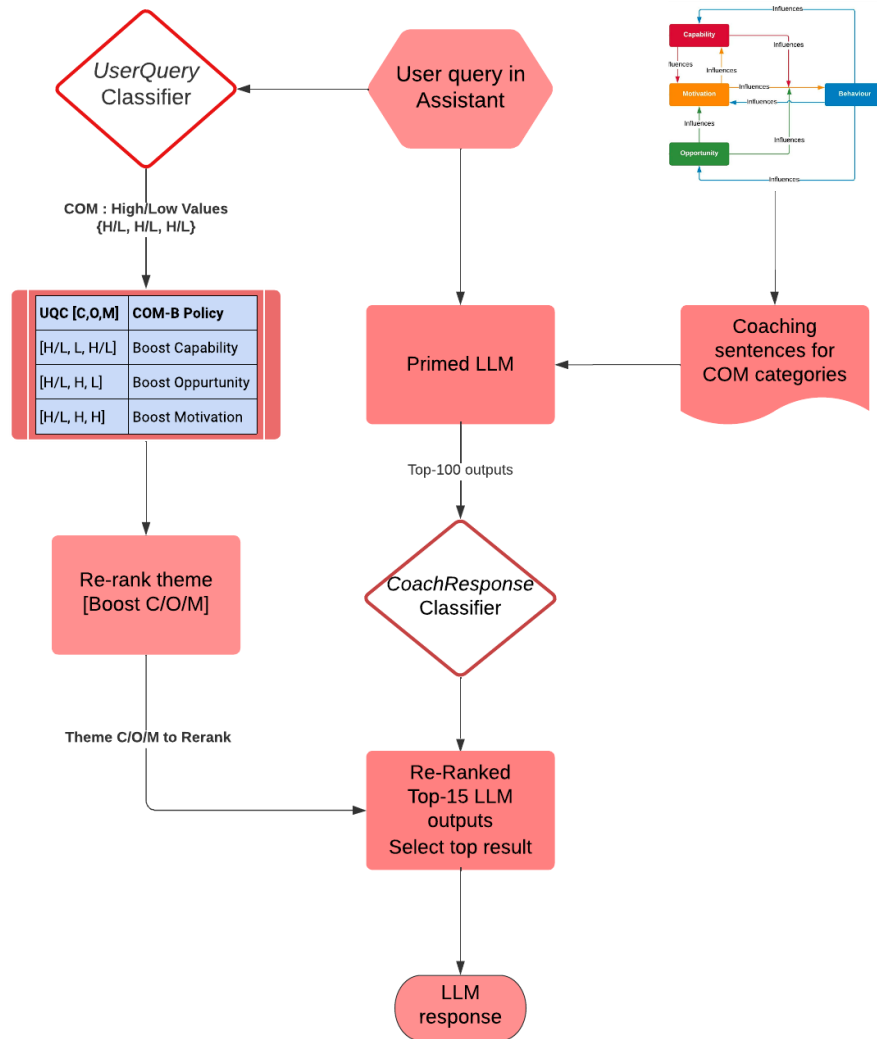

S2 Fig: Priming and BeSci infusion to LLM framework pipeline in Fit-LLM for user query input

Supplement: S2 Fig — (PDF) [file pdig.0000431.s008.pdf]
