# Supplementary material for: Infusing behavior science into large language models for activity coaching
Source: PLOS Digit Health. 2024 Apr 2;3(4):e0000431. doi: 10.1371/journal.pdig.0000431 (PMC10986996; doi:10.1371/journal.pdig.0000431)
Supplement: Attachment — Submitted filename: Response to Reviewers.pdf [file pdig.0000431.s009.pdf]

We thank the reviewers and PLoS Digital Health editorial board for giving valuable feedback on the manuscript. We have addressed all the reviewer comments

Below are revisions in the submitted documents

1. All data underlying the findings in manuscript is made fully available. These include conversations and their ratings given by raters(de-id) used in analysis, conversation snippets with LLMs, List of LLM outputs pre & post re-ranking
2. All recommendations by reviewers are addressed in the paper. The results sections include modeling parameters & effect sizes. Additional context is given below for each reviewer comment
3. While we were re-running analyses in order to add the model estimates and effect sizes to our tables, we discovered that the p-values in one table were based on the wrong set of analyses (we accidentally reported on an NP to P comparison rather than an NP to Classifier Re-Ranked comparison. We updated the table to reflect the correct analyses. The new correct comparisons are significantly more positive (in favor of the primed and re-ranked model), meaning the strength of our evidence has improved over what the reviewers initially responded to. Note that there is no change in absolute value of ratings for any of the LLM variations. Only the p-values have changed in table 8

Reviewer #1: The manuscript titled 'Infusing behavior science into large language models for activity coaching' was an interesting read, delving into a topic that seemed quite interesting and relevant. The authors explored how ideas from behavioral science may be used to enhance LLM conversations in the physical activity domain. The study is well constructed and fairly explained. However, I have some concerns which I would like the authors to modify/clarify.

1. More context to the PACE study. The authors introduce the PACE study in the introduction abruptly, without sufficient context. The explanation given on what the original study was designed for seemed a little too brief. More context to the dataset and how it was used previously would be useful.
[Done]

2. Many abbreviations are laid out without explanation (BERT, FBM, PACE). While some may be standard, it would still be important to mention what they mean.
[Done]

3. I wonder if 'qualitative review' is appropriate for the review using Likert scales. I understand what the authors mean, but I would still suggest alternative phrasing for a review that includes numerical data.
[Done] : Changed to conversational quality metrics

4. The authors mention a series of linear mixed model analyses. While the model is described briefly, I think a model equation laying out the fixed and random effects might make this much clearer. Moreover, I was not sure where the results of the model are, i.e the coefficients of each of the predictors.
[Done] : Equation and Beta coefficients added to paper

5. Post-hoc re-ranking did not seem to show significant difference from the coach primed dialogues in multiple ranking criteria. The authors could perhaps elaborate why they believe this happened/what this implies (insufficient power/why certain criteria are less likely to benefit from re-ranking).
The submitted manuscript had p-value [primed vs unprimed LAMDA] rather than [unprimed vs re-ranked LaMDA]. This is corrected in the new version of the paper with stronger statistical significance across empathy, actionability and motivational support.

We would also like to mention : The re-ranking is influenced by 3 factors : a) Accuracy of the BERT classifiers, b) number of ratings used for analysis and b) Diversity in coach primed top-k LLM responses. We see the latter (option c) as not a limiting factor as the temperature of LLM response can be increased. BERT classifier has 70% accuracy limiting correct response re-ranking, which can be improved further in future with more data. The statistical significance in findings need to be strengthened with more ratings and dialogue instances. Our findings show, LLM top-1 response may not adhere to any behavior science principles which are effective in durable behavior change. The re-raking causes the LLM response to user context and prioritization of C,O,M response introduced COM-B BeSci rules. We show statistically significant improvement in actionability since capability is of highest priority in the COM-B model and BERT model has better accuracy to aid re-ranking.

6. While p-values are useful, effect sizes might indicate how relevant the differences are. This may be more important when values are marginally significant/insignificant. It would be nice if the authors could add values for the effect size, quantified using say, the Cohen's d, for the t-tests.
[DONE] Cohed-D values added for each table

Minor edits
There seem to be quite a few minor issues scattered throughout the manuscript. A few glaring ones are noted below
1. [Done] Consistency in referring to Supplementary material, Tables etc.
2. [Done] Page 3, para 1. "consented subjects...". Consenting may be more appropriate.
3. [Done] Page 3, para 1. "consented subjects were randomized to coaches or coaches...". coaches or coaches? Rephrase for clarity
4. [Done] Page 7, para 2. A line reads simply "The (Table 5)."
5. [Done] Page 8, para 5. "Future work could trial a similar evaluation with larger groups of users engaging in
dialogue, as per [ref].", missing reference.

Reviewer #2: Summary: The goal of the authors was to integrate the COM-B principle of behavioral science into LLM through two infusion techniques: coach message priming and dialogue re-ranking. The aim was to assist users in adopting a more active lifestyle by using behavioral nudges and conversational solutions to overcome barriers.
Comments:
1. I appreciate the Authors' efforts on this paper.
Thanks

2. Authors need to justify why defining the evaluation metrics is a contribution.
Author Response : Metrics and data drive research across many fields. Our work is the first application of LLMs in fitness coaching. LLM specific metrics [column 3 in Table 3] are context agnostic and offer partial help optimizing the coaching characteristics. Contrary to LLMs, rule based coaching assistants in existing literature were task oriented and measured success based on end goals like user actions or feedback. LLMs offer conversational features closer to human coach experts which are important in building trust, relationship and influence for behavior change. Our literature survey of human fitness coaching and chatbots captures domain specific metrics to guide LLM finetuning to develop physical activity coaches and augment existing LLM metrics. These metrics guided us to finetune LLMs to reflect human conversation characteristics like empathy, actionability etc. We believe these metrics [and associated surveys to measure them] should encourage further research in the field as LLMs and coaching science evolve

3. In this paper, the authors incorporated principles and techniques from two disciplines, behavior science and computer science that serve others in other field like digital health. Integrating two disciplines is a great idea, but the reader, a specialist in one discipline, is mostly unfamiliar with the other. More shedding light on the definitions and explanations of the terms can be more beneficial to readers from different disciplines, and adding a background section might be helpful. The terms are large language model, task-oriented dialogue, behavior science framework, priming, primed LLM, and unprimed LLM.
[Done]

4. Adding a literature review section will show how the work is novel and different from the others.
[Done] First 3 paragraphs in Introduction section reviews existing lit in coaching, chatbots and LLMs

5. As mentioned in the introduction, you extend the work of PACE. Give a summary of the PACE study.
[Done]

6. Providing a screenshot or an example of one dataset record will give the reader some visualization of the dataset, especially the availability of data is restricted to some researchers.

[Done] Fig 1 is an example of conversation between user and LLM. More examples are shared in supplementary

7. Wizard of Oz protocol needs to be defined and give background about it.
[Done]
8. Some acronyms like FBM and BS need the whole sentence.
[Done]
9. Give more explanation about LaMDA.
[Done]
10. Is there any reason for keeping the model in its primary architecture without further tuning?
Tuning LLMs are expensive and ineffective for small datasets. Further, the finetuning technique was not available at the time of submission. Recently, the model was finetuned on PACE study conversation data with 520 user-coach dialogues. The model was evaluated with test data which was not used in finetuning. The bleu score of 0.6 when LLM output is compared to coach response shows better performance compared to prompt tuned and re-ranked models. We wish to continue further research in this field to finetuning both on conversation datasets and based on human feedback.

11. What interface is used for the proposed approach? Text, speech, or multi-modal?
Only text inputs

12. More elaboration about the coach message priming and dialogue ranking is needed.
[Done]

13. Text in Figure 1 needs to be clearer.
[Done]

14. Why did you use the cross entropy optimizer, not other?
Cross Entropy is the most common and effective loss function for classification problems, because it minimizes the distance between two probability distributions - predicted and actual. The BERT models are trained to classify each sentence to be one of capability, motivation or opportunity. Minimizing the cross entropy loss is equivalent to maximum likelihood estimation (MLE), which is proven to have best efficiency in optimization. The other common loss function used for classification problems and an alternative to the cross-entropy loss function is hinge loss which performs better with prior information on data characteristics like linear separability which is not applicable to our dataset.

----

6. PLOS authors have the option to publish the peer review history of their history" target="_blank">what does this mean?). If published, this will include your full peer review and any attached files.

Do you want your identity to be public for this peer review? If you choose “no”, your identity will remain anonymous but your review may still be made public.

For information about this choice, including consent withdrawal, please see our Privacy Policy.

Reviewer #1: No

Reviewer #2: No

[NOTE: If reviewer comments were submitted as an attachment file, they will be attached to this email and accessible via the submission site. Please log into your account, locate the manuscript record, and check for the action link "View Attachments". If this link does not appear, there are no attachment files.]

While revising your submission, please upload your figure files to the Preflight Analysis and Conversion Engine (PACE) digital diagnostic tool, https://pacev2.apexcovantage.com/. PACE helps ensure that figures meet PLOS requirements. To use PACE, you must first register as a user. Registration is free. Then, login and navigate to the UPLOAD tab, where you will find detailed instructions on how to use the tool. If you encounter any issues or have any questions when using PACE, please email PLOS at figures@plos.org. Please note that Supporting Information files do not need this step.

In compliance with data protection regulations, you may request that we remove your personal registration details at any time. (Use the following URL: https://www.editorialmanager.com/pdig/login.asp?a=r). Please contact the publication office if you have any questions.article (<a href="https://journals.plos.org/digitalhealth/s/editorial-and-peer-review-process#loc-peer-review-
