# Supplementary material for: Infusing behavior science into large language models for activity coaching
Source: PLOS Digit Health. 2024 Apr 2;3(4):e0000431. doi: 10.1371/journal.pdig.0000431 (PMC10986996; doi:10.1371/journal.pdig.0000431)
Supplement: Attachment — Submitted filename: Response_to_Reviewers.pdf [file pdig.0000431.s010.pdf]

We thank the reviewers and PLoS Digital Health editorial board for giving valuable feedback on the manuscript. We have addressed all the reviewer comments

Below are revisions in the submitted documents

1. Added explanation to the coefficients mentioned in equation in section 3.8
2. Added related work paragraph covering similar work in conversation agents for fitness coaching and novelty of our work.

Response to Reviewer Comments

Reviewer #1: The authors have now addressed most of the concerns I raised in the previous version. One minor addition, in section 2.8 the authors now give the equation for the models, but no explanation for what the coefficients are. I'd suggest that the authors do this during the proof stage or next submission.

[Done] : Added definition to each of the coefficients in the equation in section 2.8 (now section 3.8)

Reviewer #2: Thanks to the authors for their efforts in addressing most of the comments.
The paper needs to provide a literature review section showing how your work is novel and distinctive from other studies in the same field.

[Done] Added literature review section called Related Work [Section 2]. We have added literature related to digital health app studies and conversational agents for fitness coaching. We have also added paragraph highlighting novelty and distinctiveness from other studies in the same field.
